# Reinforcement Rate and the Balance Between Excitatory and Inhibitory Learning: Insights From Deletion of the GluA1 AMPA Receptor Subunit

**DOI:** 10.1037/xan0000336

**Published:** 2022-10

**Authors:** Joseph M. Austen, Rolf Sprengel, David J. Sanderson

**Affiliations:** 1 Department of Psychology, Durham University

**Keywords:** pavlovian conditioning, mice, conditioned inhibition, summation test, prediction error

## Abstract

Conditioned responding is sensitive to reinforcement rate. This rate-sensitivity is impaired in genetically modified mice that lack the GluA1 subunit of the AMPA receptor. A time-dependent application of the Rescorla–Wagner learning rule can be used to derive an account of rate-sensitivity by reflecting the balance of excitatory and inhibitory associative strength over time. By applying this analysis, the impairment in GluA1 knockout mice may be explained by reduced sensitivity to negative prediction error and thus, impaired inhibitory learning, such that excitatory associative strength is not reduced during the nonreinforced periods of a conditioned stimulus. The article describes a test of the role of GluA1 in inhibitory learning that requires summing of the associative strengths of cues presented in compound. Mice were trained on a feature negative discrimination of the form A+/AX–. GluA1 knockout mice acquired the discrimination to a similar extent as controls. The inhibitory properties of cue X were verified in a summation test that included a control for nonassociative, external inhibition. The performance of GluA1 knockout mice was similar to that of controls. However, in line with previous findings, GluA1 deletion impaired the precision of timing of conditioned responding. These results provide further evidence that impaired sensitivity to reinforcement rate is not a consequence of impaired inhibitory learning. The results may more readily fit with accounts of rate sensitivity that propose that it reflects encoding of temporal and numeric information rather than being a consequence of changes in associative strength over time.

The duration of a conditioned stimulus (CS) affects the strength of conditioned responding such that short-duration CSs elicit stronger responding than long duration CSs (Austen et al., 2021; Gibbon et al., 1977; Harris & Carpenter, 2011; Holland, 2000; Lattal, 1999). An error correction learning rule, such as the Rescorla–Wagner model (Rescorla & Wagner, 1972) implemented iteratively over time, provides a potential account of this CS duration effect (e.g., Harris et al., 2015). Thus, for a normal delay conditioning procedure in which a cue is reinforced at its termination, the strength of learning increases during periods of reinforcement and decreases during the periods of nonreinforcement prior to the occurrence of reinforcement. This process results in an asymptotic level of learning that is determined by the ratio of periods of reinforcement to nonreinforcement. This time-dependent application of an error correction learning rule results in learning being sensitive to the rate at which reinforcement occurs over cumulative exposure. Although the Rescorla–Wagner model (Rescorla & Wagner, 1972) is often depicted as a trial-based account of learning, it was necessary for the model to assume that changes in associative strength occur over time. The model was able to accommodate temporal parameters in, for example, Rescorla’s (1968) contingency experiments, by representing the passage time in the intertrial interval as a continuous series of trials equal in duration to the length of the CS. As mentioned above, by extending this idea to time periods shorter than the trial duration, such that a CS trial can be considered a series of microtrials, it is possible to account for the effects of trial duration on learning (Harris et al., 2015).

The time-based application of the Rescorla–Wagner learning rule (Rescorla & Wagner, 1972) was necessary to account for Rescorla’s (1968) experiments, using the ‘truly random control procedure’. In those experiments rats received presentations of electric shocks that occurred in the presence and absence of a tone at particular rates. When the rate of shock in the presence of the tone was greater than in its absence, rats showed conditioned suppression of lever pressing to the tone. When the rate of shocks was equal in the presence and absence of the tone, rats failed to show conditioned responding. The Rescorla–Wagner model (Rescorla & Wagner, 1972) accounts for this finding by assuming not only that associative strength is gained and lost over time depending on the presence/absence of reinforcement, but also that conditioned stimuli compete for associative strength with contextual stimuli that are present during the procedure. The Rescorla–Wagner learning rule is shown in Equation 1. When the rule is implemented in a time-dependent manner changes in associative strength (V) on a given moment for a particular stimulus (A) are equal to the discrepancy between λ, the maximum amount of associative strength supported by the unconditioned stimulus (US), and the sum of the associative strength of all cues present (X) multiplied by learning parameters determined by the salience of the CS (α) and US (β). The discrepancy between λ and the current total associative strength, the prediction error, can be positive (i.e., the US was not fully predicted) or negative (i.e., the US was expected to a greater extent than its actual occurrence). Because changes in associative strength for a particular CS are determined by the current total associative strength of all cues present rather than that of the CS only, this means that a CS will lose associative strength when the total associative strength exceeds λ. A consequence of this is that a CS that fails to signal an increase in reinforcement above the background rate of reinforcement will eventually achieve zero associative strength. Furthermore, cues that signal a decrease in reinforcement rate acquire negative associative strength. Therefore, inhibitory conditioning in which a CS signals the absence of an otherwise expected reinforcement reflects the sum of inhibitory (negative) and excitatory (positive) associative strength.

$$\Delta {\text{V}}_{\text{A}}=\alpha_{\text{A}}.\beta \left(\lambda -\Sigma {\text{V}}_{\text{x}}\right).$$ 1

Recent work examining the neural basis of time-sensitive learning provides a means for testing assumptions of the time-based application of the Rescorla–Wagner learning rule. Across a series of experiments, genetically modified mice that lack the GluA1 subunit of the AMPA receptor, a key mediator of synaptic plasticity in brain regions implemented in learning and memory, failed to show sensitivity to reinforcement rate but instead were sensitive to the number of times that a CS was reinforced (Austen et al., 2021). For example, in Experiment 1 of Austen et al. (2021), mice were trained with two CSs that differed in reinforcement rate by virtue of differences in their duration (10 s vs. 40 s). Wild-type (WT), control mice, responded more to the 10-s cue than the 40-s cue. Mice lacking the GluA1 AMPA receptor subunit (GluA1^–/–^), however, responded at a similarly high rate to both cues. Indeed, response rates (and difference scores that indicated responding above the response rate for a nonreinforced cue) for the 40 s cue were significantly higher in GluA1^–/–^ mice than WT mice. In Experiment 3 of Austen et al., mice were similarly trained with a 10-s and 40-s cue, but now extra nonreinforced trials for the 10-s cue were added such that the 10 s cue was reinforced on only 25% of trials. This resulted in matching the overall reinforcement rates of the 10 s and 40 s cues. WT mice now failed to show a significant difference and responded at similarly low rate to the two cues, consistent with the hypothesis that response levels are sensitive to the rate of reinforcement. GluA1^–/–^ mice showed a similarly high level of responding to the two cues. Indeed, response rates were significantly higher for the two cues in GluA1^–/–^ mice than in WT mice. Therefore, GluA1^–/–^ mice failed to show sensitivity to cue duration when the 10 s and 40 s cues were continually reinforced in Experiment 1 and they were insensitive to the effect of partially reinforcing the 10 s cue in Experiment 3. Austen et al. concluded that GluA1 plays a selective role in learning about reinforcement rate. Indeed, the results of further experiments reported by Austen et al. suggested that GluA1^–/–^ mice are more sensitive to manipulations of the number of CS–US pairings than reinforcement rate.

The Rescorla–Wagner learning rule (Rescorla & Wagner, 1972), as described previously, can be applied in a time-dependent manner in order to account for sensitivity of learning to reinforcement rate as manipulated by cue duration and probability of reinforcement per trial. This analysis of reinforcement rate sensitivity provides a potential account of the impairment in GluA1^–/–^ mice. Thus, rate-sensitivity requires incrementing associative strength during periods of reinforcement and reducing associative strength during periods of nonreinforcement. An impairment in reducing associative strength during periods of nonreinforcement results in learning that increments with each reinforcement up to a maximum level but fails to reflect the reinforcement rate of the cue. This was the pattern of performance that was observed with GluA1^–/–^ mice and could be simulated by reducing the learning rate parameter for learning in the absence of the US to zero (Austen et al., 2021; see the online supplemental materials for Austen et al., 2021). Therefore, GluA1 deletion may impair the ability to reduce learning when an otherwise expected reinforcement is omitted. If this is the case, then GluA1 deletion should impair learning that reflects inhibitory associative strength. This hypothesis was tested using an extinction learning procedure (Austen et al., 2021). GluA1^–/–^ mice, however, showed normal levels of extinction.

The purpose of the present study was to provide a more stringent test of inhibitory learning in GluA1^–/–^ mice. While extinction learning reflects a form of inhibition, it does not necessarily require changes in associative strength that depend on the summed associative strength across cues as described by the Rescorla–Wagner model (Rescorla & Wagner, 1972). Here, we used the feature negative discrimination procedure in order to examine inhibitory learning that reflects the summed associative strength across cues. Mice received reinforced (+) trials of A+ and nonreinforced (–) trials of the compound AX–. In order to learn the discrimination mice must learn that the compound signals the absence of reinforcement despite A being reinforced when presented alone. According to the Rescorla–Wagner model such learning occurs because cue X acquires negative associative strength. Consequently, the total associative strength of the compound is less than that of A alone. In addition to A+ and AX– trials, mice also received B–, BY–, C+ and Z+ trials throughout training. Trial types B–, BY– and C+ were included in order that, after training, a summation test could be administered to verify the inhibitory properties of cue X by assessing its ability to reduce responding to another cue with which it had not previously been paired. In the summation test, mice were presented with the compound CX. If responding to the compound is less than to C alone then this would suggest that the combined associative strength of CX is less than C and therefore X has reduced the level of responding that cue C would otherwise elicit. It is possible, however, that responding to the compound CX may be low simply due to a decrement in generalization of learning from C to the novel compound CX. In order to control for this form of external inhibition, mice also received presentations of the compound CY. Prior to the summation test, cue Y had received similar training to X in that it was presented in compound with another cue (B) and was nonreinforced. In contrast to X, cue Y did not signal a reduction in the expected rate of reinforcement. Because cue B did not signal reinforcement when presented alone, the associative strength of Y remains at zero when nonreinforced in compound with B. Therefore, although any level of generalization decrement between cue C and the two compounds was matched between the compounds, if X had acquired inhibitory associative strength, then responding to CX should be lower than to CY. Because cues X and Y were from the same modality (either visual or auditory) and were both nonreinforced, another cue from the same modality was used for reinforced Z+ trials in order to decrease generalization between the cues.

In addition to examining acquisition of the feature negative discrimination and performance in the summation test, the distribution of responding within trials was also examined. Previous work has found that the timing of responding is less precise in GluA1^–/–^ mice (Austen et al., 2021; see the online supplemental materials, Figure S2 in Austen et al., 2021). Responding increased across the duration of the cue, but this increase was significantly steeper in WT mice compared with GluA1^–/–^ mice indicating that a higher proportion of responses were made toward the end of the cue, close in time to the delivery of the US. Furthermore, using the peak procedure, it was found that the distribution of responding around the expected occurrence of reinforcement was significantly broader for GluA1^–/–^ mice than WT mice. Consistent with impaired precision of timing in GluA1^–/–^ mice, GluA1 deletion impaired inhibition of delay in trace conditioning, in which responding is increasingly withheld during the initial portions of a CS (Sanderson et al., 2017). Within the present experiment, assessment of timing of conditioned responding served several purposes. A deficit in timing would provide positive verification of the effect of GluA1 deletion. Furthermore, impaired encoding of temporal durations generally, as potentially measured by the timing of conditioned responding, may underlie a deficit in reinforcement rate sensitivity.

## Method

Mice were GluA1*^–/–^* (16 female, six male) and WT (eight female, seven male) age-matched littermates, bred in the Life Sciences Support Unit, Durham University (see Zamanillo et al., 1999 for details of genetic construction, breeding and subsequent genotyping). The mice were originally derived from the 129S2svHsd and C57BL/6J/OlaHsd strains and have been subsequently backcrossed onto the C57BL/6J line. Mice were housed in groups of one to eight in a temperature-controlled holding room on a 12-hr light–dark cycle (light period: 8 a.m. to 8 p.m.). Mice were approximately 14 to 18 weeks old at the start of testing and mean weight was 21.6 g (range = 16.6–31.8g). For several days prior to the start of testing, the weights of the mice were reduced by restricting access to food and they were maintained at 85% of their free-feeding weights throughout the experiment. Mice had ad libitum access to water in their home cages. All procedures were conducted under the Home Office U.K. Project License No. PPL 70/7785 and approved by Durham University Animal Welfare and Ethical Review Board.

### Apparatus

A set of eight identical operant chambers (interior dimensions: 15.9 × 14.0 × 12.7 cm; ENV-307A, Med Associates), enclosed in sound-attenuating cubicles (ENV-022V) were used. The operant chambers were controlled by Med-PC IV software (SOF-735). The side walls were made from aluminum, and the front and back walls and the ceiling were made from clear Perspex. The chamber floors each comprised a grid of stainless-steel rods (.32 cm diameter), spaced .79 cm apart, running perpendicular to the front of the chamber (ENV-307A-GFW). A food magazine (2.9 × 2.5 × 1.9 cm; ENV-303M) was situated in the center of one of the sidewalls of the chamber, into which sucrose pellets (14 mg, TestDiet) could be delivered from a pellet dispenser (ENV-203-14P). An infrared beam (ENV-303HDA) across the entrance of the magazine recorded head entries at a resolution of .1 s. A fan (ENV-025 °F) was located within each of the sound-attenuating cubicles and was turned on during sessions, providing a background sound level of approximately 65 dB. Auditory stimuli were provided by a white noise generator (ENV-325SM) that outputted a flat frequency response from 10 to 25,000 Hz at 80 dB, a clicker (ENV-335M) that operated at a frequency of 4 Hz at 80 dB, and a pure tone generator (ENV-323AM) that produced a 2,900 Hz tone at 80dB. Visual stimuli were a 2.8 W house light (ENV-315M) mounted at the top of the wall opposite the food magazine, and two LEDs mounted to the left and right above the food magazine (ENV-321M). The left LED was programmed to flash twice per second (.25 s on/.25 s off) and the right LED provided constant illumination.

### Procedure

#### Training

Mice received 16 sessions of discrimination training, one session per day. Each session consisted of A+, AX–, B–, BY–, C+ and Z+ trials. There were four trials per trial type per session. Each trial lasted 10 s and reinforced trials terminated with the presentation of a sucrose pellet. The interval between trials was 120 s (CS offset to CS onset). The order of trials was random with the constraint that for each block of 12 trials there were two trials of each trial type. For approximately half the mice within each sex within each genotype, cues A, B and C were auditory, and X, Y and Z were visual. The opposite was true for the remaining mice. For mice for which cues A and B were auditory, A and B were the clicker and tone and X and Y were the house light and flashing LED. The allocation of auditory cues (clicker and tone) to A and B and the allocation of visual cues (house light and flashing LED) to X and Y was counterbalanced, in a 2 (auditory cue) × 2 (visual cue) factorial design, within each sex, within each genotype in an approximate manner given the numbers of mice for each of these subgroups. For mice for which cues A and B were visual and X and Y were auditory, the cues were counterbalanced in a similar manner. If cues A and B were auditory then C was white noise and Z was the constant LED. The opposite was true for mice for which A and B were visual.

#### Summation Test

Twenty-four hours after the last day of training, mice received a summation test session. The compounds CX and CY were presented six times each and were nonreinforced. In addition, mice received 12 trials of C+, which continued to be reinforced with the presentation of a pellet. The order of trials was random with the constraint that each block of four trials consisted of two C+ trials, one CX trial and one CY trial. All other details were the same as during training.

### Data and Statistical Analysis

The number of magazine entries during trial types was recorded and expressed as responses per minute (RPM). Timing of conditioned responding was examined by fitting linear slopes to the number of responses per second, which were normalized for the overall number of responses for each mouse. Acquisition data were analyzed using multifactorial analysis of variance (ANOVA) and linear slopes were analyzed using an independent sample *t* test. Mice that failed to show greater levels of responding to A+ than AX– in the latter half of training were excluded from the analysis of the summation test. This criterion resulted in six mice (two female and one male WT and three female GluA1^–/–^) being excluded and 12 WT (six female, six male) and 19 GluA1^–/–^ (13 female, six male) remaining. Response rates for CX and CY were analyzed using a 2 (trial-type) × 2 (genotype) ANOVA. In addition, we also report suppression ratios in which response rates to the two compounds are expressed as a proportion of the total response rate to the compound and C+. These ratios potentially increase the sensitivity of the comparison between CX and CY by reducing the influence of variability in response rates to C+ (e.g., Bonardi et al., 2010).

## Results

### Training

**Figure 1 fig1:**
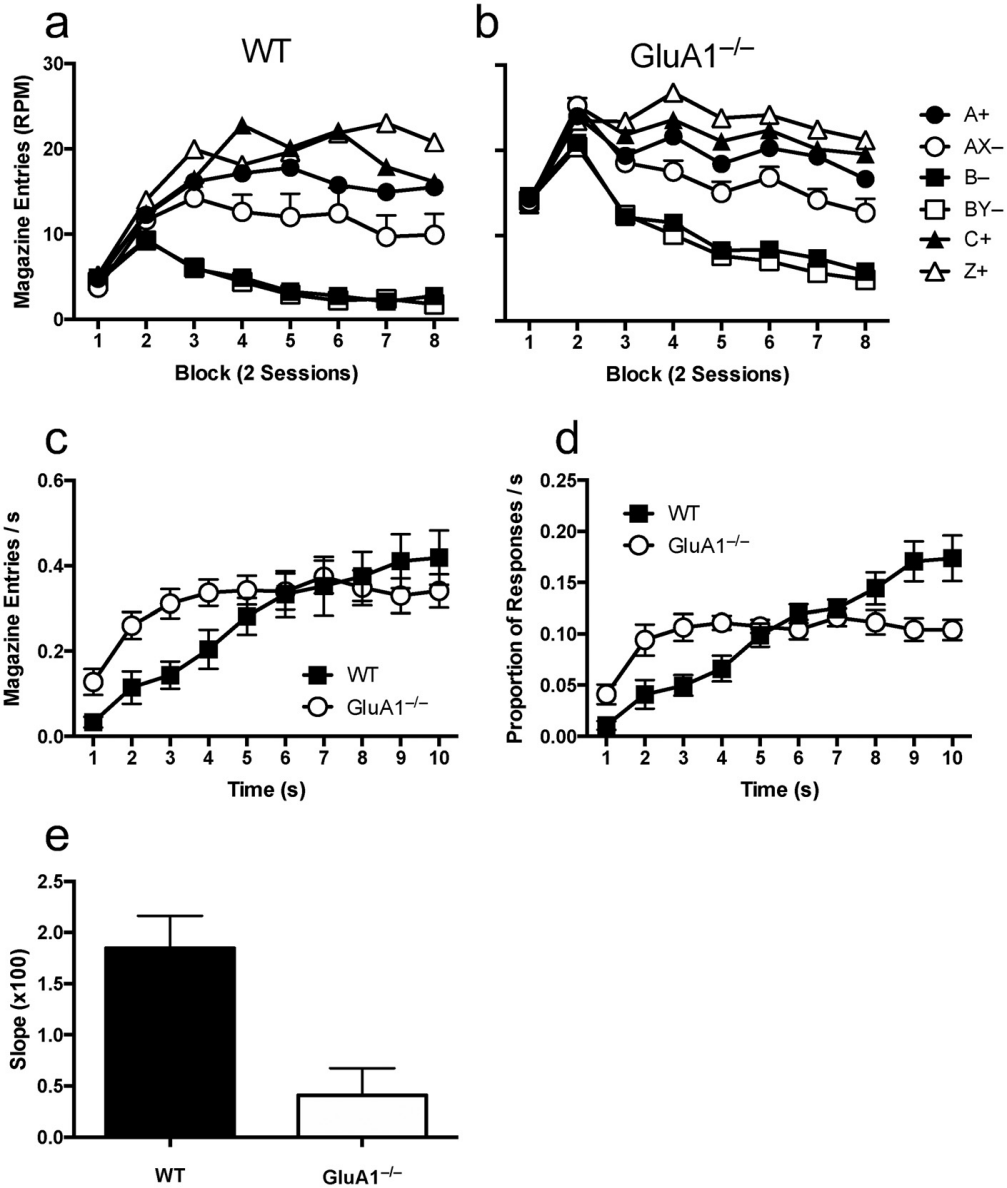
Performance During Acquisition of the Feature Negative Discrimination *Note*. Acquisition for wild-type (Panel a) and GluA1^–/–^ mice (Panel b) is shown over blocks of two sessions. Response rates are show as responses per minute (RPM). Error bars indicate the standard error of the mean difference between response rates for A+ and AX– trial types. Responding as function of time within the A+ trial type is shown collapsed across trials in the latter of half of training in Panel c. The mean normalized rates of responding reported in Panel c are shown in Panel d. Error bars indicate standard error of the mean. Slopes fitted to the normalized response rates for within A+ trials are shown in Panel e. Error bars indicate standard error of the mean.

Responding across blocks of two sessions is shown in Figure 1, Panels a and b. Across blocks, mice came to respond more to reinforced trial types than nonreinforced trial types. A 2 (genotype) × 2 (trial type) × 8 (block) ANOVA comparing response rates for A+ and AX– trials showed a significant effect of trial type, *F*(1, 35) = 15.79, *MSE* = .357, *p* < .001, η^2^ = .31, 90% CI [.11, .47] and block, *F*(7, 245) = 7.75, *MSE* = .163, *p* < .001, η^2^ = .18, 90% CI [.09, .23] and significant interaction of factors, *F*(7, 245) = 4.73, *MSE* = .022, *p* < .001, η^2^ = .12, 90% CI [.04, .16]. GluA1^–/–^ mice responded significantly more than WT mice, *F*(1, 35) = 4.93, *MSE* = 1.18, *p* = .03, η^2^ = .12, 90% CI [.01, .29] and this effect significantly interacted with block, *F*(7, 245) = 3.46, *MSE* = .073, *p* = .01, η^2^ = .09, 90% CI [.02, .13]. Simple main effects analysis of the genotype by block interaction revealed that GluA1^–/–^ mice responded at a significantly higher level than WT mice in the first two blocks, smallest *F*(1, 35) = 9.87, *p* = .003), but not thereafter, largest *F*(1, 35) = 3.12, *p* = .09. The Trial Type × Genotype and Trial Type × Block × Genotype interactions were not significant (*F*s < 1).[Fig fig1]

In order to determine whether discrimination of A+ and AX– had reached asymptote by the end of training, the Trial Type × Block interaction was analyzed by comparing the difference in response rates to the trial types over blocks. The interaction was simplified by subtracting responses rates for AX– from response rates for A+ and then post hoc analyses of the effect of block were conducted using least square difference. It was found that the difference in response rates between trial types was significantly lower in Blocks 1 through 3 than in subsequent blocks (e.g., Block 3 was significantly lower than Blocks 5, 7, and 8; *p* < .05), but Blocks 4 through 8 did not significantly differ from one another (smallest *p* = .09). Therefore, there was no significant increase in the extent of the discrimination between A+ and AX– from Block 4 onward.

The distribution of responding as a function of time within the A+ trial type, collapsed across Blocks 5 through 8, in the latter half of training, is shown in Figure 1, Panel c. Whereas responding plateaued at approximately halfway through the trial for GluA1^–/–^ mice, WT mice showed a monotonic increase over time. To compare the relative steepness of the increases in responding within the duration of the trial, slopes were fitted to response rates per second that were normalized for overall response rates. The mean normalized responses rates per second are shown in Figure 1, Panel d and the mean slopes for WT and GluA1^–/–^ mice are shown in Figure 1, Panel e. A *t* test comparing slopes across genotypes showed that slopes were significantly steeper for WT mice than for GluA1^–/–^ mice, *t*(35) = 3.47, *p* = .001, *d* = 1.16, 95% CI [.45, 1.87].

### Summation Test

**Figure 2 fig2:**
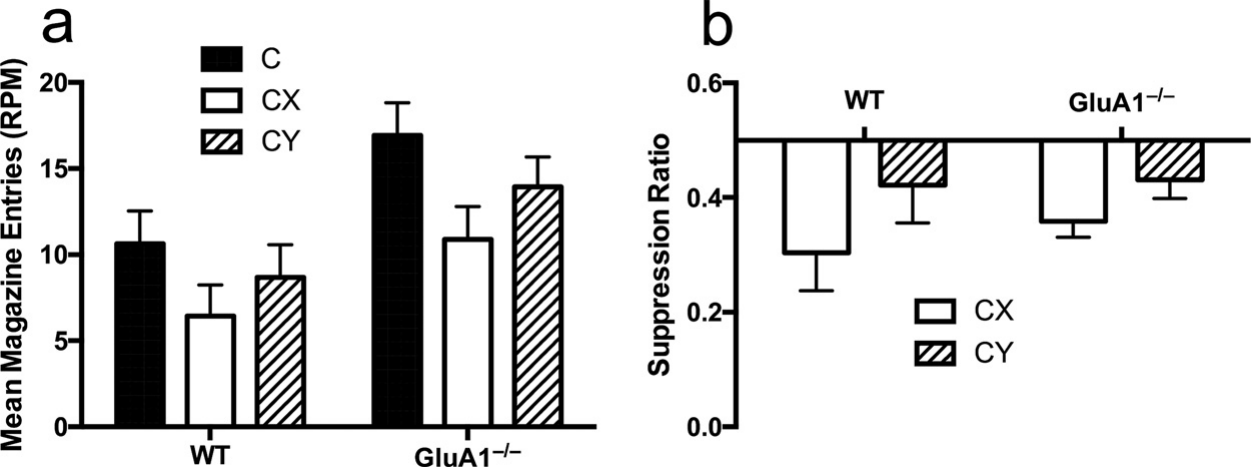
Performance in the Summation Test *Note*. Panel a shows the response rates for C, CX and CY trials. Response rates are shown as responses per minute (RPM). Panel b shows responding to the compounds CX and CY as a ratio of total responding to the compound and to C. Ratios less than 0.5 indicate suppression of responding to the compound relative to C. Error bars indicate standard error of the mean.

Six mice (two female and one male WT and three female GluA1^–/–^) were excluded from the analysis of the summation test due to failing to show greater responding to A+ than AX– in the latter half of training. The remaining number of mice per genotype were 12 WT (six female, six male) and 19 GluA1^–/–^ (13 female, six male). The rates of responding to CX, CY and C+ trials are show in Figure 2, Panel a. Mice showed reduced levels of responding to the compounds CX and CY compared with C+ trials and levels of responding to CX were lower than for CY. In order to test whether response rates were significantly lower for CX than CY a 2 (trial type: CX, CY) × 2 (genotype) ANOVA was conducted. It was found that there was a significant effect of trial type, *F*(1, 29) = 4.29, *MSE* = .029, *p* = .047, η^2^ = .12, 90% CI [.0008, .31]. Although GluA1^–/–^ mice responded more than WT mice the effect of genotype failed to reach significance, *F*(1, 29) = 4.12, *p* = .052. The interaction of factors was not significant (*F* < 1). Analysis of the suppression ratios (shown in Figure 2, Panel b) showed that ratios were significantly lower for CX than for CY, *F*(1, 29) = 5.72, *MSE* = .134, *p* = .023, η^2^ = .16, 90% CI [.01, .35]. There was no significant effect of genotype or interaction of factors (*F*s < 1). The ratios for CX and CY indicate the relative reduction in responding to the compounds compared with C+ trials. An analysis of responding on C+ trials alone demonstrated that GluA1^–/–^ mice responded more than WT mice, *t*(29) = 2.21, *p* = .035, *d* = .81.[Fig fig2]

## Discussion

The purpose of the experiment was to test whether the cause of impaired sensitivity to reinforcement rate in GluA1^–/–^ mice is a deficit in inhibitory learning that reflects calculation of summed associative strength of cues within a trial. Overall, GluA1^–/–^ mice showed similar levels of acquisition of the feature negative discrimination and similar performance on the summation test suggesting that conditioned inhibition learning is not impaired by GluA1 deletion. This demonstrates that GluA1 is not necessary for learning as a consequence of negative prediction error generally and it is not necessary for error calculations that take into account the summed associative strength of all cues present on a trial. The results may suggest that sensitivity to reinforcement rate is not simply a consequence of balancing changes in excitatory and inhibitory learning.

GluA1^–/–^ and WT mice showed similar levels of discrimination between A+ and AX– trials. Although GluA1^–/–^ mice initially showed higher levels of responding overall at the start of training, they came to respond at a similar level to WT mice for the subsequent majority of training. Both groups showed greater responding to A+ than AX– and the extent of this difference was similar in both groups. Three mice from each genotype failed to respond more to A+ than AX– in the latter half of training suggesting that they had failed to learn the discrimination. This number as a proportion of each genotype was lower in GluA1^–/–^ mice than in WT mice. Therefore, there was no evidence of an impairment in feature negative discrimination learning in GluA1^–/–^ mice.

It is possible that further training may have led to greater discrimination between A+ and AX–, which may have then revealed a difference between the genotypes. Analysis of the Trial Type × Block interaction, however, revealed that the extent of discrimination between A+ and AX– did not significantly increase from block four onward, which may suggest that performance had reached an asymptotic level. Some studies of feature negative discrimination learning have used procedures in which AX– trials are more frequent than A+ trials (e.g., Cole et al., 1997) based on the hypothesis that inhibitory learning proceeds more slowly than excitatory learning (see Rescorla, 2002, for a discussion). In the current experiment, the number of A+ and AX– trials was equal. It is possible that an increase in the proportion of AX– trials relative to A+ trials may lead to greater discrimination, which may improve the sensitivity of the test of conditioned inhibition between the genotypes. Further work is needed to determine whether GluA1 deletion would affect acquisition of the feature negative discrimination under conditions that would facilitate greater discrimination between A+ and AX– in control animals.

The summation test assessed whether, as a consequence of the feature negative discrimination training, cue X had acquired inhibitory properties that would transfer when paired with a different excitatory cue, C, which had been reinforced on separate trials. Given that a reduction in responding to the novel compound, CX, compared with C, may be caused by external inhibition in addition to conditioned inhibition, responding to CX was compared with another compound, CY, for which the level of external inhibition was matched. Cue Y had previously been trained in a similar manner to X except that when it was nonreinforced in the compound BY there was no negative prediction error that would result in inhibition. This is because cue B was also nonreinforced when presented alone such that reinforcement was not expected during the nonreinforced presentations of BY. The rate of responding to CX was significantly lower than to the compound CY, thus ruling out the possibility that the reduction in responding to CX compared with C was due only to generalization decrement caused by the novel combination of cues. The fact that responding was lower to CX than CY in the summation test suggests that X had acquired inhibitory properties as a specific consequence of negative prediction error during nonreinforced presentations of AX in the training phase.

GluA1^–/–^ mice and WT mice both showed weaker responding to CX than CY and the extent of this difference was numerically similar in the two genotypes. GluA1^–/–^ mice did, however, tend to respond more overall compared with WT mice during the summation test and responding on C+ trials did significantly differ between genotypes. This may complicate comparisons between levels of summation performance between the genotypes. Discrimination ratios that normalized for the rate of responding on C+ trials failed to demonstrate a difference between the genotypes. This suggests that the reduction in responding to CX and to CY as a proportion of the rate of responding to C was similar between GluA1^–/–^ and WT mice. Although caution should be taken when making comparisons of the extent of the difference in responding to CX and CY at different points on the response measure scale, at the very least, there was no evidence of a reduced summation effect in GluA1^–/–^ and WT mice. Further work is needed to determine whether GluA1 deletion would affect performance when responding across genotypes is compared across a similar baseline.

GluA1 deletion failed to impair performance of conditioned inhibition as measured by the feature negative discrimination procedure and the summation test. We have similarly failed to find an effect of GluA1 deletion on extinction of Pavlovian conditioning (Austen et al., 2021). Whereas extinction learning does not require learning based on the summed associative strength of cues, inhibitory learning as a consequence of the feature negative discrimination does. Therefore, the present results provide further evidence that GluA1 is not necessary for the loss of associative strength during nonreinforcement and is not required for calculations of prediction error based on summation of associative strength across cues.

The fact that GluA1 deletion failed to impair inhibitory learning suggests that our previous finding that GluA1 deletion impairs sensitivity to rate information cannot be explained by impairments in inhibitory learning and a lack of sensitivity to nonreinforcement. Therefore, the time-dependent application of the Rescorla–Wagner learning rule in which the effects of GluA1 deletion can be modeled by reducing the learning rate in the absence of reinforcement down to zero (see the online supplemental materials in Austen et al., 2021) fails to account for the lack of effect of GluA1 deletion on conditioned inhibition.

The results are in contrast to those of a study that found that GluA1 deletion impaired inhibitory conditioning that occurred as a consequence of trace conditioning (Sanderson et al., 2017). Mice were trained with two auditory cues. One cue signaled reinforcement after a trace interval (either 4 s or 8 s) and the other cue was nonreinforced. WT mice initially showed excitatory conditioning with the trace conditioned cue but after prolonged training they came to respond less to the trace conditioned cue than to the nonreinforced cue, suggesting that the trace conditioned cue had acquired inhibitory properties similar to an inhibition of delay effect (Rescorla, 1967). GluA1^–/–^ mice also initially showed excitatory conditioning with the trace conditioned cue, but in contrast to WT mice they continued to respond more to the trace conditioned cue than the nonreinforced cue. Therefore, GluA1 deletion impaired the switch to withholding responding to the trace conditioned cue that developed with prolonged training. The fact that responding to the trace conditioned cue in WT mice eventually became significantly lower than to the nonreinforced cue suggests that the reduction in responding to the trace conditioned cue was a specific consequence of its temporal arrangement with reinforcement. Furthermore, mice trained with a cue that immediately terminated in reinforcement did not reduce responding over prolonged training. The current results demonstrate that GluA1 deletion does not impair inhibitory conditioning per se. Instead, the impairment in inhibitory conditioning observed with prolonged training of trace conditioning (Sanderson et al., 2017) suggests that GluA1 is necessary when temporal factors are the cause of the inhibition.

Real-time models of learning, such as temporal difference learning (Sutton & Barto, 1981) and Wagner’s (1981) Sometimes Opponent Process (SOP) model, have attempted to account for the role of time in a number of procedures. These models are, however, unlikely to account readily for the role of GluA1 in reinforcement-rate sensitivity without making several assumptions about the relevant parameters. One issue is that these accounts do not necessarily anticipate that learning will be sensitive to reinforcement rate. Thus, rats and mice show similar rates of responding to cues that are matched for cumulative reinforcement rate but differ in CS duration and probability of reinforcement (Austen et al., 2021; Harris et al., 2015). Models such as temporal difference learning and Wagner’s SOP model share similarities with the Rescorla-Wagner model, but they contain extra free parameters that are used to account for the role of time in learning. Thus, the temporal difference learning model assumes that future rewards are discounted to the extent that they are delayed such that responding will be weaker to longer duration cues than shorter duration cues. Wagner’s SOP model contains parameters for the decay of short-term memory that results in reduced processing over long duration cues. While these models, in principle, should be able to account for some rate sensitivity effects, they may do so only at an extreme cost of adopting highly specific parameter discounting or decay values that may not be reasonably applied to other factors that determine learning.

We have proposed that GluA1 deletion may impair rate-sensitivity due to a role of GluA1 in timing (Austen et al., 2021). GluA1^–/–^ mice show a significantly broader peak in responding around the expected time of reinforcement in the peak procedure suggesting a reduced precision in timing ability (Austen et al., 2021; see Experiment 8 described in the online supplemental materials of Austen et al., 2021). Similarly, the increase in responding across the duration of a trial is less steep in GluA1^–/–^ mice compared with WT mice (Austen et al., 2021; see the online supplemental materials of Austen et al., 2021). This was also observed in the feature negative discrimination training in responding on A+ trials (see Figure 1, Panel e); WT mice showed steeper increases in responding than GluA1^–/–^ mice, indicating that a higher proportion of their responses were made close to the time of reinforcement. Furthermore, as described previously, we have observed qualitatively different patterns of responding within trials between GluA1^–/–^ and WT mice that were a specific consequence of a trace interval procedure (Sanderson et al., 2017). In the case of rate-sensitivity, this impaired precision in timing in GluA1^–/–^ mice may result in an inability to discriminate between cues with different trial durations or different cumulative durations between reinforcements. This analysis of GluA1 assumes that rate sensitivity is a consequence of symbolic encoding of CS durations and number of reinforcements such that rate information can be derived. This is in keeping with models such as rate estimation theory (Gallistel & Gibbon, 2000) and the temporal coding hypothesis (Molet & Miller, 2014) that posit that animals explicitly represent temporal durations and that memory of temporal intervals is part of the content of learning.

An issue that remains is that GluA1 deletion failed to impair learning about nonreinforced trials in the feature negative procedure and in extinction, but it impaired learning about nonreinforced trials during acquisition of responding to a partially reinforced cue (Austen et al., 2021). A potential explanation for the difference between extinction learning and acquisition of partial reinforcement is that acquisition is sensitive to reinforcement rate over cumulative exposure to a cue irrespective of how the exposure is structured in terms of trial duration and probability of reinforcement per trial (Harris et al., 2015), but, in contrast, extinction is sensitive to the number of reinforced trials irrespective of trial duration (Chan & Harris, 2017; Harris, 2019; Harris & Andrew, 2017). Therefore, while GluA1 deletion may have impaired sensitivity to the duration of nonreinforced exposure during acquisition of responding to the partially reinforced cue, it spared sensitivity to the number of nonreinforced trials during extinction.

The results failed to demonstrate that GluA1 is necessary for conditioned inhibition suggesting that the role of GluA1 in rate-sensitivity is unlikely to be in determining balances in gains and losses of associative strength over cumulative exposure to a CS. Therefore, the results are not consistent with the time-based application of the Rescorla–Wagner model that has been used to account for the role of relative reinforcement rates in the contingency effect (Rescorla, 1968; Rescorla & Wagner, 1972). We now consider an alternative account that, rather than rate-sensitivity being a consequence of temporal processes that affect associative strength, rate-sensitivity reflects encoding of temporal and numeric information which can be used to derive estimations of rate. Nonetheless, further work is needed in order to verify whether GluA1 deletion fails to impair conditioned inhibition when inhibition learning is acquired to a greater strength and when the effect of baseline responding can be controlled.

Genetic manipulation of the GluA1 subunit of the AMPA receptor has revealed striking dissociations between particular psychological processes. In addition, to its role in reinforcement rate-sensitivity, GluA1 is necessary for short-term but not long-term memory expression in tests of relative familiarity (Reisel et al., 2002; Sanderson & Bannerman, 2012; Sanderson et al., 2007, 2009, 2011a, 2011b; Schmitt et al., 2003). By identifying the psychological role of GluA1 we have been able to use GluA1 deletion as a tool for testing theoretical accounts of learning. The advance of neural manipulations provides additional means by which to dissociate particular psychological processes that might not be dissociated through behavioral manipulations alone.
